# High stability microwave discharge ion sources

**DOI:** 10.1038/s41598-022-06937-7

**Published:** 2022-02-23

**Authors:** L. Neri, L. Celona

**Affiliations:** INFN-Laboratori Nazionali del Sud, via S. Sofia 62, 95123 Catania, Italy

**Keywords:** Design, synthesis and processing, Applied physics, Information theory and computation, Plasma physics, Techniques and instrumentation

## Abstract

A new plasma heating mechanism for Microwave Discharge Ion Sources (MDIS) was discovered. Unprecedented beam stability was observed during the commissioning of the Proton Source for the European Spallation Source (PS-ESS) where several thousand source configurations were tested using a custom software tool. Data analysis and plasma simulation revealed that the new behaviour is generated by a completely new plasma heating schema activated by a precise magnetic configuration peculiarity. The stability showed in this configuration, denominated High Stability Microwave Discharge Ion Source (HSMDIS), is excellent and the emittance of the produced beam is lower than produced by standard MDIS configuration. High linearity between power and beam current was observed making easier the use of the source. This new mode of operation can be easily implemented in all existing sources.

## Introduction

Electron Cyclotron Resonance (ECR) ion sources are widely used today for several purposes ranging from the production of ion beams for research to industrial applications. ECR heating is one of the most reliable ways to heat the plasma in an ion source^1^. When the circular orbit, of the electrons in a magnetic field, have the same revolution frequency as the microwave, the electron can be accelerated/decelerated along the entire orbit, which is equal to say along the entire microwave period. The acceleration is spatially located in correspondence of the resonant magnetic field and the amplitude of the acceleration is proportional to the electromagnetic field orthogonal to the magnetostatic field. Outside of this region, the synchronism is lost, the electrons are alternatively accelerated and decelerated with no net energy gain. The ECR condition is expressed by the well-known formula:

$$\omega_{RF} = \frac{{q_{e} B_{ECR} }}{{m_{e} }}$$

where *ω*_*RF*_ is the microwave angular frequency, *q*_*e*_ the electron elementary charge, *B*_*ECR*_ the magnetic field at the resonance, and *m*_*e*_ the electron mass. This condition cannot be achieved in the entire ECR ion sources plasma chamber volume, but there are always a wide variety of magnetic field configurations compatible with ECR condition.

Two kinds of magnetic configurations are commonly used. The first used in the so-called ECR sources^1–3^, is optimized to achieve high plasma confinement time and consequently high charge state ion production. It is obtained by superimposing a solenoidal field and a hexapolar field. In this magnetic configuration, the ECR value is reached in a closed surface located at the centre of the plasma chamber. The disadvantage of high confinement time is the low extracted current value. The relation in between this quantity is expressed by the phenomenological relations^4^:

$$I \propto \frac{N}{T}$$

$$q_{opt} \propto \log N\omega T$$

where the *I* is the ion density, *N* is the plasma density, *T* is the plasma confinement time and *q*_*opt*_ is the charge state giving the maximum ion current.

The latter magnetic configuration is used in 2.45 GHz ECR sources, called also Microwave Discharge Ion Source^5–8^, designed to produce a high current of a single charged ion beam. Typical ion beams are H^+^, D^+^, H_2_^+^, while current ranges from one to more than a hundred milliamperes. High beam current values are reached by using only a solenoidal field in a low axial confinement configuration. The standard magnetic field configuration used in this type of source is described in^5^. The ECR magnetic field condition is achieved at the two ends of the plasma chamber, the microwave injection side and the beam extraction side, while in the central part of the plasma chamber, the magnetic field value is slightly higher than the ECR value.

This paper presents the discovery of a new magnetic field configuration that allows the MDIS source to produce higher stability and lower emittance beam. The discovery was done during the commissioning of the high-intensity Proton Source for the European Spallation Source (PS-ESS)^9–12^ at INFN-LNS. Plasma simulations, here presented, show that the corresponding plasma heating schema and plasma dynamics are completely different compared to the standard configuration. For those reasons, we named this new mode of operation High Stability Microwave Discharge Ion Source (HSMDIS). In the following, the experimental characterization and the plasma simulation study are presented to disclose how this new heating schema works and what are the differences with the standard one.

## Experimental setup

The source PS-ESS was designed to achieve two goals. The first is to satisfy all the stringent beam requirements of the ESS project and the latter to give the possibility to explore new source configurations. Our innovation was needed and reinforced by the knowledge that ESS requirements were never achieved before by standard MDIS sources. A simple optimization procedure starting from standard MDIS configuration would not be enough. For that reason, a high degree of freedom was inserted in the design of the source magnetic system^13^, making it able to produce a large variety of magnetic field configurations. To enable the test of such a large variety of magnetic configurations several innovations were inserted in the design of the source. The source was designed to be capable to sustain also worst configurations. The microwave injection system was designed to be able to achieve a good plasma matching for all possible plasma densities and to also support the total power reflection in case of no plasma ignition^14^. The extraction system was designed to reduce as much as possible the production of high voltage sparks. Moreover, the extraction system was designed to sustain beam extraction with a current double of what was requested. The control system electronics was designed to operate without faults in presence of high voltage sparks occurring in case of non-stable beam production. At last, an objective, exhaustive and extensive characterization strategy was adopted to test all possible source configurations.

Usually, the standard optimization strategy was based on the manual change of the source parameters and the visual inspection of the Faraday cup and/or Current Transformer on an oscilloscope. The output was the mean beam current value and a subjective evaluation of produced beam stability. This optimization methodology is also limited by the number of configurations that can be tested by manually setting the source parameters. In the PS-ESS development strategy, a high-level control system^15^ was developed to send, sequentially, a large set of configuration parameters to the source and acquire from the beam diagnostic equipment several numerical quantities quantitatively describing the source performances. Everything was designed to run without any human interaction, to get a fast characterization (only 10 s for each source configuration), and to produce a complete and objective quantitative analysis. Fifty-five thousand source configurations were considered of interest and tested. Each one was characterized by acquiring statistics for eighty consecutive beam pulses, 26 parameters were evaluated, and stored, to describe the extracted beam and the source status. Finally, two waveforms at 1 ms/s were saved from the Faraday Cup (FC) and AC Current Transformer (ACCT) electronic acquisition boards. The great amount and the high quality of the collected data make possible the mapping of the regions where the source performs differently. The quality of the description was increased by an accurate choice of the set of parameters identifying the magnetic configuration: three values of the on-axis magnetic field, respectively at 0 mm, 35 mm and 84 mm from the injection flange. The analysis of the source performance, in this space of parameters, shows a clear correlation with the value of the magnetic field. The magnetic field in these three locations together with the injected microwave power and the amount of H_2_ gas fluxed in the plasma chamber (directly proportional to the pressure inside the plasma chamber^16^) represent the five parameters identifying the source configuration. The fifty-five thousand source configurations tested range in the limits reported in Table 1.

**Table 1:** Ranges of source configuration parameters tested during the commissioning.

Parameter	Min	Max
Magnetic field at 0 mm	795 G	1015 G
Magnetic field at 35 mm	515 G	1395 G
Magnetic field at 84 mm	235 G	1995 G
H_2_ flow	2 SCCM	5 SCCM
RF power	550 W	650 W

Considering the cylindrical symmetry of the plasma chamber and the magnetic system, we can deduce that the magnetic field goes from the field value on the axis to the field value on the walls almost linearly and independently from the angular coordinate. Therefore, by showing the magnetic field profile on-axis and on the plasma chamber radius, we can have a clear quantitative representation of the magnetic field over the entire plasma chamber volume. It must be remarked that previously, the identification of the magnetic configuration^5^ was restricted to considerations regarding only on-axis magnetic field profile.

**Figure 1:**
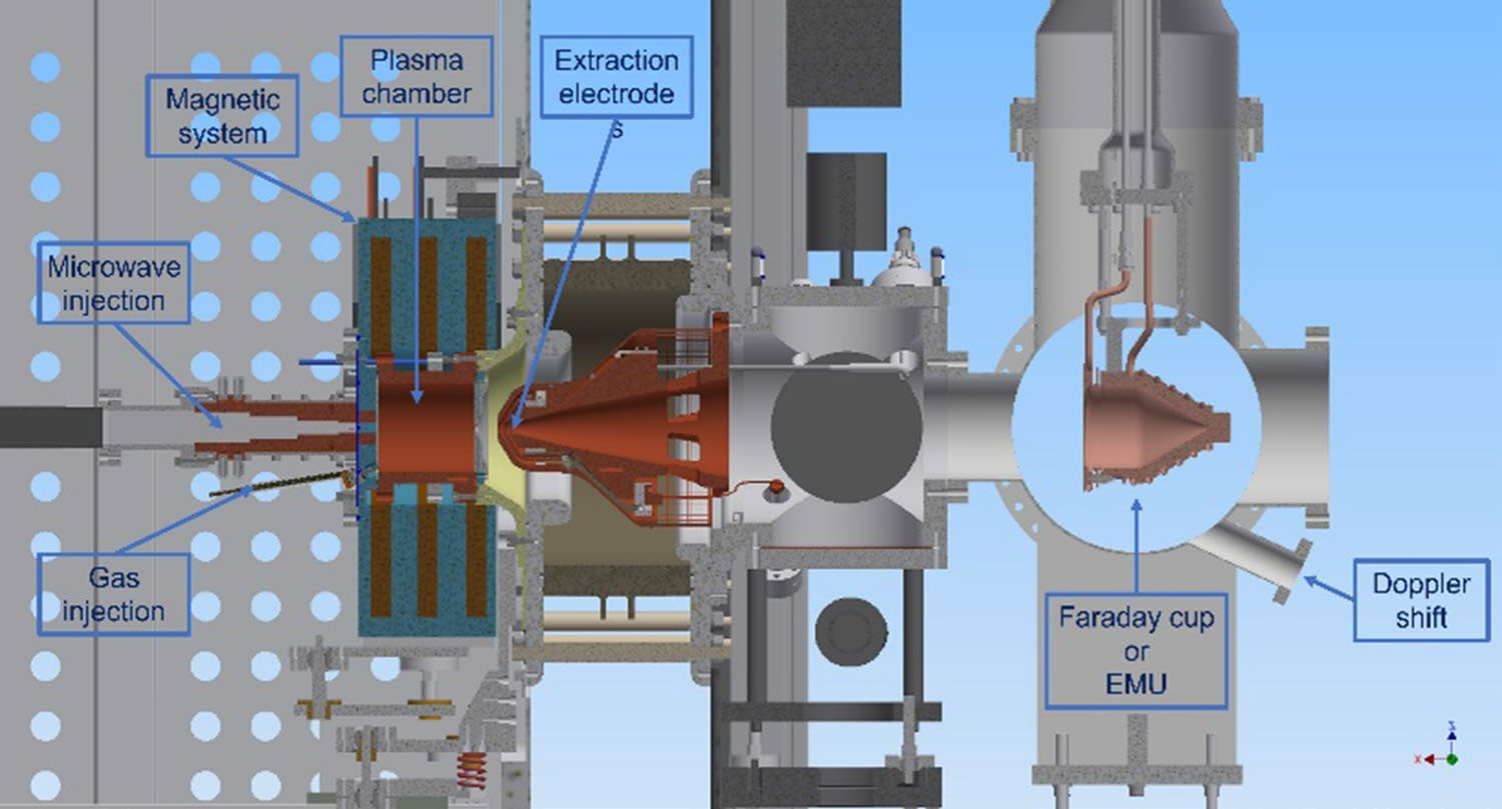
A half sectional view of the source and the first part of the LEBT (picture generated with Autodesk-Inventor).

The experimental setup used in the first step of PS-ESS commissioning is reported in Fig. 1 where a cross-section view of the source and the beam diagnostic chamber is presented. It shows the cylindrical plasma chamber 10 cm long and 10 cm in diameter, the three coils magnetic system, the microwave injection line, the gas injection, the extraction system, part of the high voltage platform housing the source, and the diagnostics chamber with the FC, the position of the Emittance Measurement Unit (EMU) and Doppler Shift measurement unit. The total beam current extracted from the source is measured with the ACCT installed on the high voltage cable providing the current needed to bring the voltage of the platform up to the operating value of 75 kV. The measurement of this current is equivalent to the measurement of the total positive beam current extracted from the source plus the negative current of back streaming electrons from the LEBT. This latter current never exceeded the value of 4 mA.

### Simulation strategy

**Figure 2:**
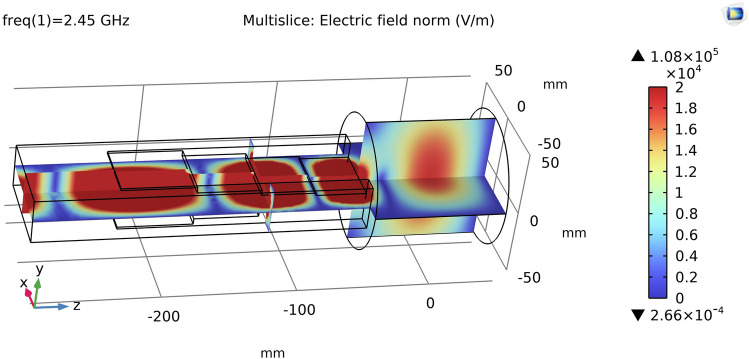
Microwave electric field amplitude (picture generated with Comsol-Multiphysics).

The simulation strategy adopted in the past 10 years^17,18^ has been focused to single phenomenon acting on the source and its relative contribution to the system, without pointing to the objective to build a complete and predictive ion source simulation tool. The present paper is a perfect example of how a not predictive simulation tool can help in the understanding of how a different magnetic configuration can significantly change the source behaviour. The following analysis is carried out by using only the first step of the stationary-PIC strategy^19^ developed by our group. The simulation code used in this work, developed in Matlab language, has the only objective to visualize the energy gain of electrons moving inside the electromagnetic field in presence of two different magnetic configurations. The three-dimensional magnetic field map was evaluated by applying a rotation to the field computed by using a two-dimensional axisymmetric Comsol Multiphysics model with the geometry of the magnetic system and the ferromagnetic elements of the plasma chamber (mesh size of 1 mm). The electromagnetic microwave field map (Fig. 2) is computed with a Comsol Multiphysics model of the empty plasma chamber cavity and the matching transformer using a mesh size of 3 mm. Frequency domain mode and direct solver have been used. The motion of 2E6 electrons has been computed, starting from a temperature distribution of 0.1 eV, with a time step of 5E−12 s by using the Boris method^20^:

$$\vec{x}_{k + 1} = \vec{x}_{k} + \Delta t \cdot \vec{v}_{k + 1/2}$$

$$\vec{v}_{k + 1/2} = \vec{u}^{\prime} + q^{\prime} \cdot \vec{E}_{k}$$

With:

$$\vec{u}^{\prime} = \vec{u} + (\vec{u} + (\vec{u} \times \vec{h})) \times \vec{s}$$

$$\vec{u} = \vec{v}_{k - 1/2} + q^{\prime} \cdot \vec{E}_{k}$$

$$\vec{h} = q^{\prime} \cdot \vec{B}_{k}$$

$$s = 2 \cdot \vec{h}/(1 + h^{2} )$$

$$q^{\prime} = \Delta t \cdot \frac{q}{2 \cdot m}$$

$$\vec{E}_{k} = real\left( {\vec{E}_{RF} (\vec{x}) \cdot e^{{\left( {i \cdot \omega_{RF} \cdot k \cdot \Delta t} \right)}} } \right)$$

$$\vec{B}_{k} = real\left( {\vec{B}_{RF} (\vec{x}) \cdot e^{{\left( {i \cdot \omega_{RF} \cdot k \cdot \Delta t} \right)}} } \right) + \vec{B}_{s} (\vec{x})$$

where the position vector at time step k ($\vec{x}_{k}$) and the velocity vector at the time step k − 1/2 ($\vec{v}_{k - 1/2}$) are updated by using the particle charge $q$ and mass $m$, the time step $\Delta t$, the magnetostatic field map $\vec{B}_{s} (\vec{x})$ and the electromagnetic field maps $\vec{E}_{RF} (\vec{x})$ and $\vec{B}_{RF} (\vec{x})$. Coulomb collisions are considered with background plasma density maps uniformly distributed in the entire plasma chamber volume with a density of 1E17 particles per cubic meter and electron temperature of 6 eV^21^. The collisions are computed by considering the following three relaxation times (chapter ten of^22^).

Slowing down time:

$$\tau_{s} = - \frac{v}{{\left\langle {\Delta v_{||} } \right\rangle }}$$

Deflection time:

$$\tau_{d} = \frac{{v^{2} }}{{\left\langle {\left( {\Delta v_{ \bot } } \right)^{2} } \right\rangle }}$$

Energy exchange times:

$$\tau_{e} = \frac{{E^{2} }}{{\left( {\Delta E} \right)^{2} }}$$

where velocity and energy of each electron are compared with the mean values of the plasma. The computation ends when 95% of the electrons are lost in the walls, approximately after 1E−6 s. The quantities extracted from the simulation are evaluated by summing the contribution of each electron for each time step of their entire life and the result is stored in different output maps with 0.6 mm grid size. The computational time needed for each of the two simulations run here presented is of about 30 h by using an I9-7980xe CPU.

### Standard MDIS magnetic configuration

**Figure 3:**
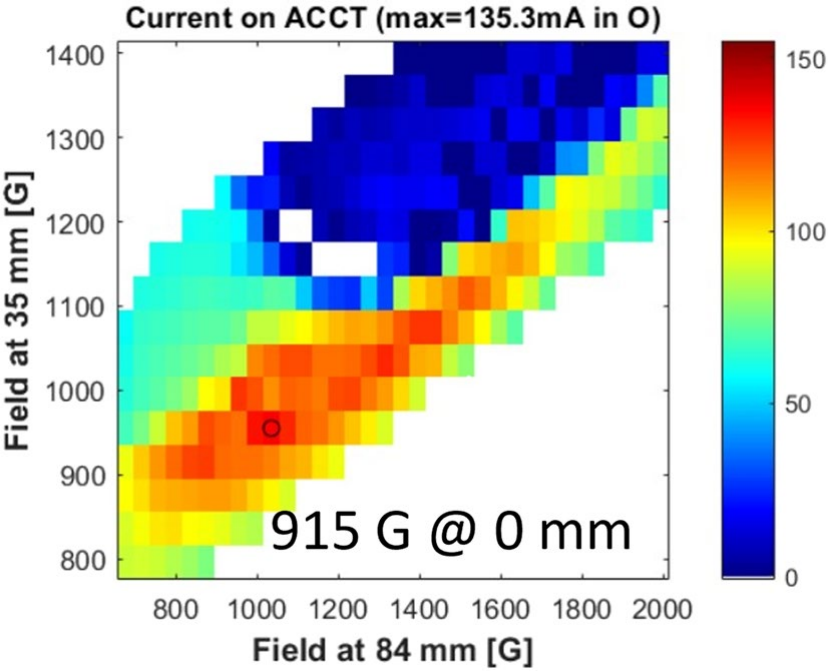
ACCT current measured around the standard MDIS configuration; colour scale proportional to the mean produced beam current in mA.

The first evidence found with the analysis of the 55 thousand source configurations tested, was that close to the standard MDIS magnetic configuration^5^ there is a well-defined region where the source shows a high current production. This area is evident in Fig. 3 where ACCT current is shown as a function of the magnetic field at 35 mm and 84 mm, these data are the subset of measurement taken with 915 Gauss at 0 mm from the injection flange, 600 W of injected microwave power and 3.0 Standard Cubic Centimetres per minute (SCCM) of H_2_ gas flux. Figure 3 clearly shows the key role played by the source magnetic field. The maximum high-intensity production (marked with a circle) is in correspondence of 955 Gauss at 35 mm and 1035 Gauss at 84 mm.

**Figure 4:**
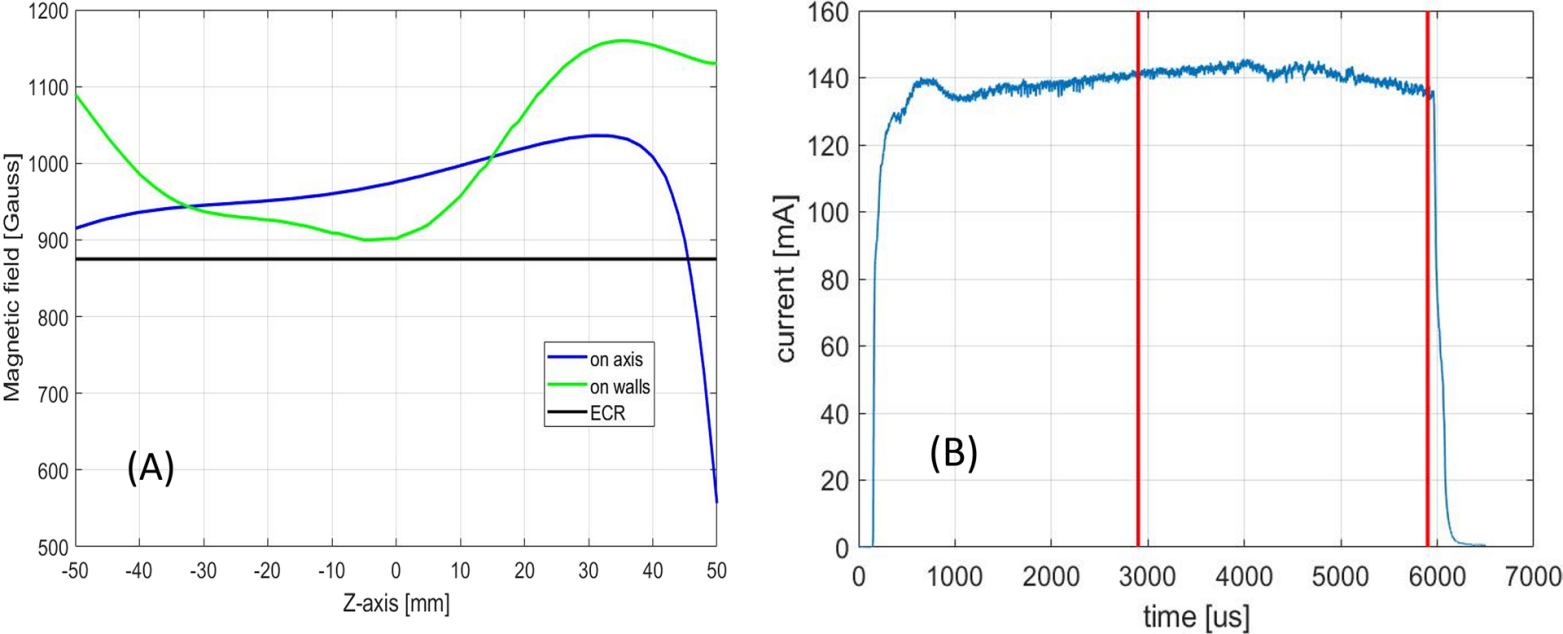
(**A**) Magnetic field configuration at the centre of the high current region; (**B**) Correspondent beam pulse shape produced: only the beam produced within the red lines is transmitted to the accelerator.

The magnetic configuration at the centre of the high current region is shown in Fig. 4A. The on-axis magnetic field profile shows a value slightly over the ECR value close to the microwave injection (located at z = − 50 mm), an almost flat region at the centre of the plasma chamber and an abrupt magnetic field drop in correspondence of the extraction (located at z = 50 mm) where an ARMCO ferromagnetic shielding is placed. The on-walls magnetic field profile is always over the ECR value meaning that there are no other ECR regions in the plasma chamber. This magnetic configuration is close to what is the standard MDIS magnetic configuration, presented in 1991 by Taylor and Wills^5^. MDIS was developed to produce high-intensity beams, it is so evident that the standard MDIS magnetic configuration is close to the magnetic configuration that produces the highest intensity beam. All existing MDIS machines work with a magnetic configuration close to the standard one, small variations are due to peculiarities of each magnetic system and evidence of relative better beam stability production.

The beam pulse produced by the configuration at the centre of the high current region is shown in Fig. 4B. The ESS project requires 2.86 ms of constant beam pulse with a repetition rate of 14 Hz, pulse to pulse stability of 3.5% and flat-top stability of 2%. Therefore, it was chosen to turn ON the source for 6 ms: the first 3 ms to reach a stable beam current condition and the last 3 ms to send a stable beam to the accelerator. The first 3 ms are blocked by the LEBT Chopper^23,24^. Considering the flat-top beam pulse of high-current magnetic configuration, eighty consecutive pulses were considered, the mean beam current measured is 135.3 mA (as reported in Fig. 3), while the mean beam current of the pulse presented in Fig. 4B is 141 mA. This discrepancy is due to low stability between pulses, furthermore, inside the flat-top, the beam pulse is not constant and noisy. In conclusion, this configuration permits to reach beam-pulse average current well above the ESS requirements, while the stability does not satisfy the ESS requirement. This low-quality beam stability may not represent a showstopper for some applications, however, for the ESS project it represents an increase of beam loss in the acceleration chain and an increase of uncertainty in the neutron flux amount sent to the experiments. Therefore, it cannot be accepted as almost all the magnetic configurations in the region of high current production. Sometimes, going slightly away from the centre of the high current region a more stable configuration can be found, but the emittance of the produced beam^25,26^ is higher than the ESS requirement.

### MDIS plasma simulation

**Figure 5:**
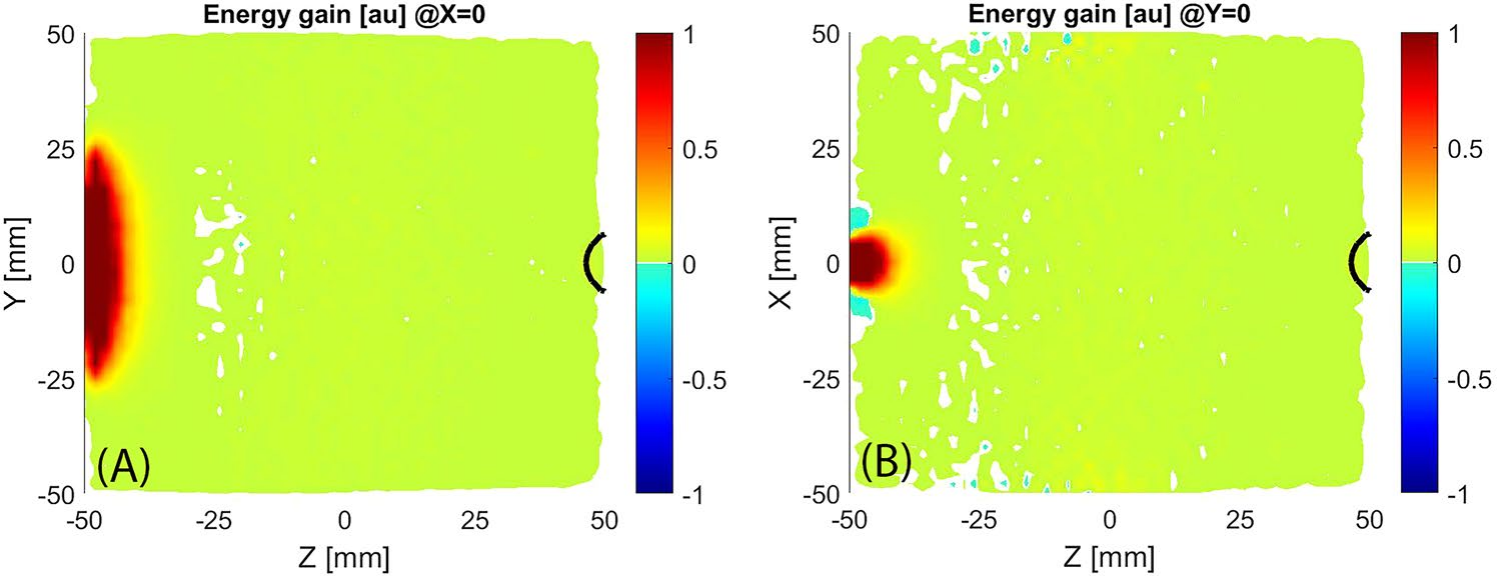
X = 0 and Y = 0 slice views of the energy gain distribution, black line marks the ECR region.

The electromagnetic field produces the acceleration or deceleration of electrons during their trajectory. Summing the energy variation (positive or negative) to which each electron has been subjected passing through the cells of the domain, it is possible to identify the region where the electrons usually gain or lose energy. Plasma simulation of the standard MDIS magnetic configuration shows a plasma heating region close to the microwave injection flange (Fig. 5). A high energy gain is observed in correspondence with the microwave injection aperture due to the high amplitude of the electric field of this region. This is not obvious because the magnetic field in this region is over the ECR value (915 Gauss). The rectangular shape of the microwave injection aperture can be recognized on the X = 0 and Y = 0 slice views. The little energy gain, uniformly distributed in the rest of the plasma chamber, is due to energy exchange, by Coulomb collisions, with the background plasma that was initialized with 6 eV temperature distribution. No energy gain is observed in the ECR region close to the extraction hole due to a very low intensity of the microwave electric field in this region.

**Figure 6:**
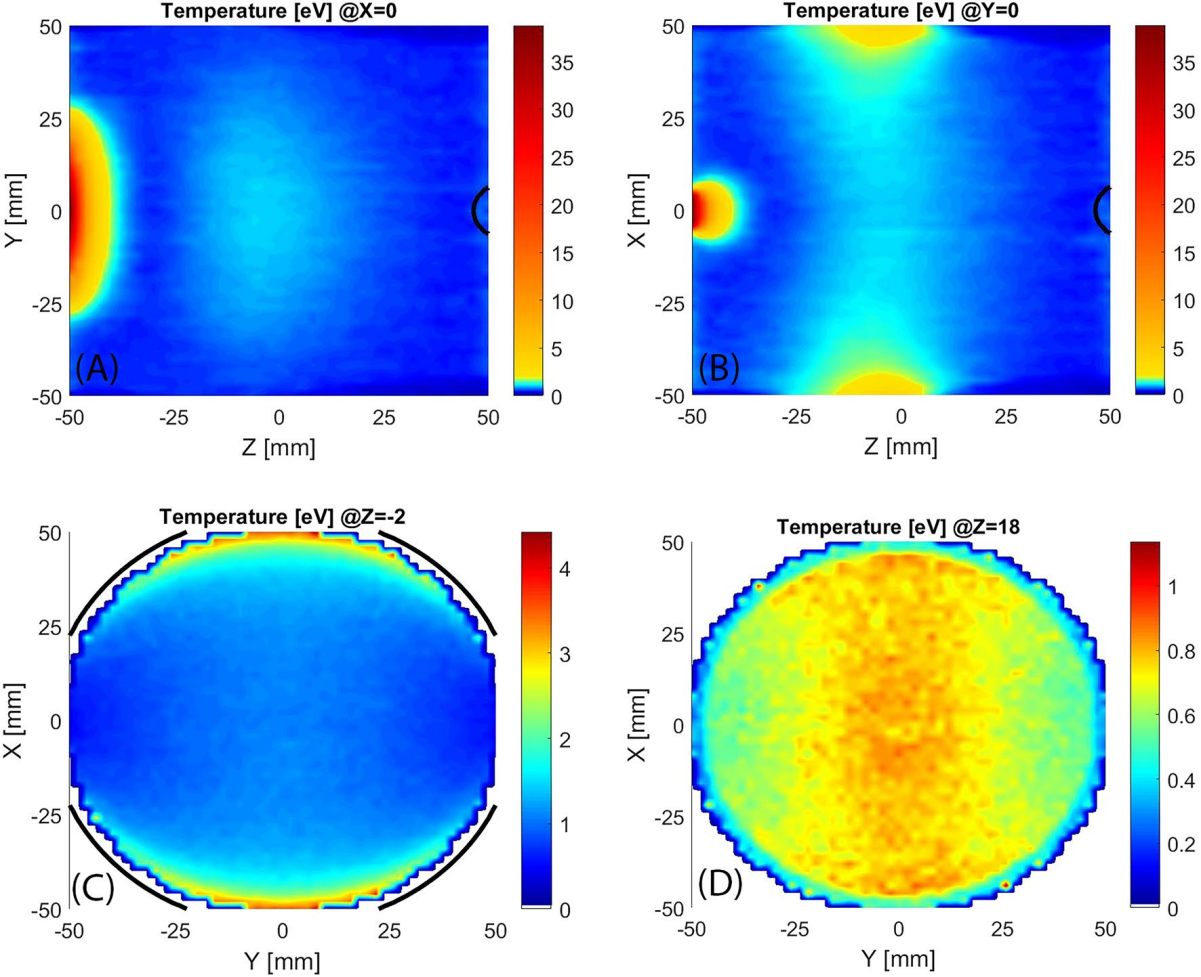
Longitudinal slices (**A**,**B**) and transversal slices (**C**,**D**) of evaluated electron temperature spatial distribution, black line marks the ECR region.

An evaluation of the Electron Energy and Spatially Distribution Function (EESDF) may be carried out by storing the energy of electrons passing through each cell. With a Maxwellian fit, it is possible to extract the temperature spatial distribution (Fig. 6). In the longitudinal sections (6A and 6B) the colour scale is divided into two parts: the first part (from blue to green) is used for temperatures ranging from 0 eV to 5% (1.9 eV) of the maximum evaluated temperature (38 eV), while the latter part (from yellow to red) is used from 5 to 100%. In the two transversal views (6C and 6D), obtained at two different longitudinal positions (Z = − 2 mm and Z = 18 mm) the colour scale is uniform because there is only a warm population. Longitudinal views show a relatively high temperature in correspondence with the microwave adsorption region and aggregation of warm electrons in the centre of the plasma chamber with an hourglass shape oriented as the microwave electric field.

**Figure 7:**
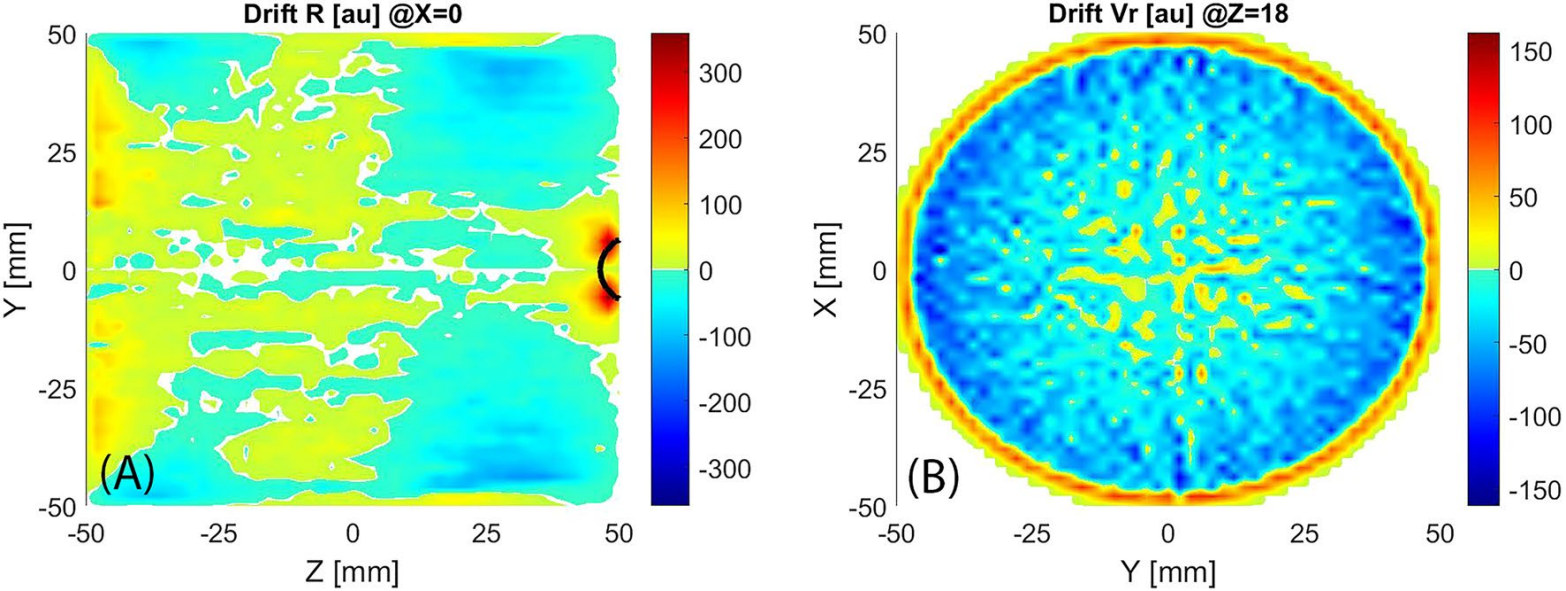
A longitudinal (**A**) and a transversal (**B**) slice of the radial drift velocity, black line highlights the ECR region.

Summing the velocity of electrons passing through each cell of the domain, it is possible to reveal the presence of drift velocity (Fig. 7). The radial component is the most important because shows the tendency of electrons to be lost in the walls (positive radial drift velocity, colour scale from yellow to dark red) or vice versa (negative radial drift velocity, blue colour scale part) to be focused on the centre of the plasma chamber. Two confinement regions are located close to the cylindrical walls, the region closer to the extraction (10 mm < z < 45 mm and R > 25 mm) is wider and stronger compared to the other one (− 45 mm < z < − 20 mm and R > 35 mm). In these regions, the electrons are not lost in the walls enabling the formation of high-density plasma and high current production. Two not very intense deconfinement regions are in correspondence of the extraction system (35 mm < z < 50 mm and R < 20 mm) and the microwave injection (− 50 mm < z < − 35 mm and R < 40 mm).

**Figure 8:**
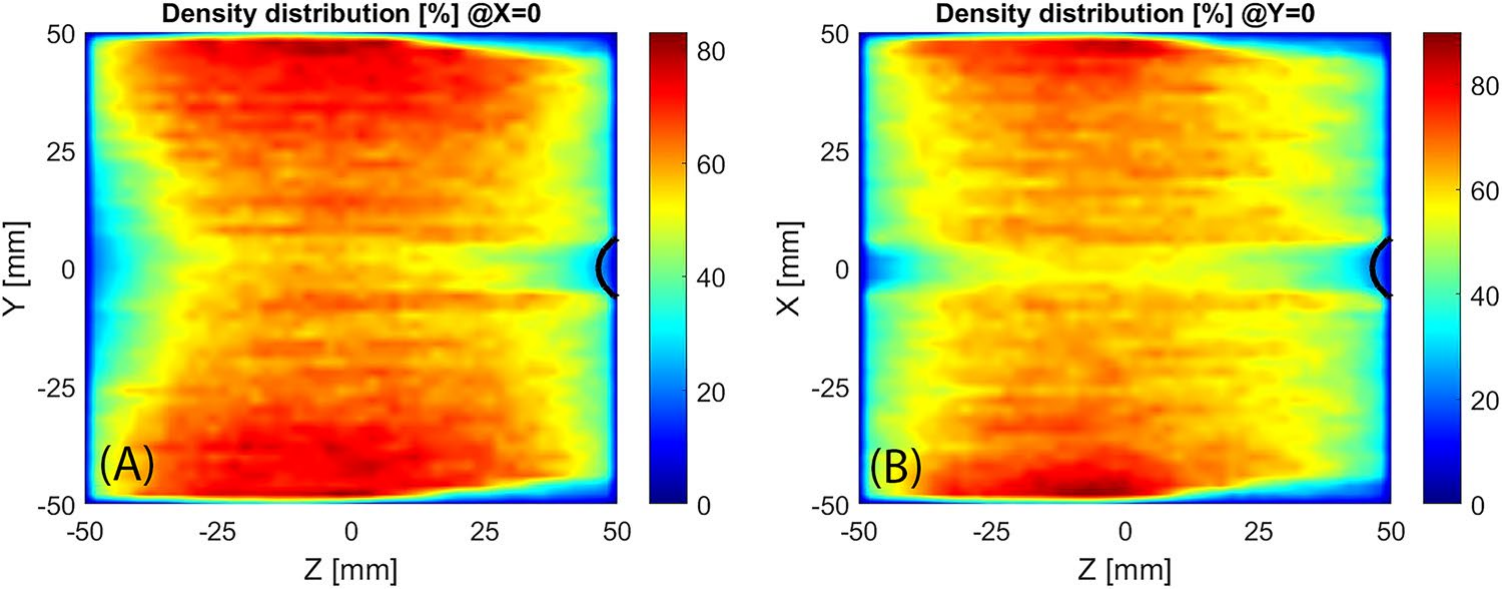
Longitudinal sections of electron density, black line marks the ECR region.

The evaluation of the electron density distribution may be carried out by summing the time spent from electrons inside each cell of the domain. The simulation does not permit to quantitatively evaluate the density, only the spatial distribution can be considered for the analysis, for this reason, the density is shown (Fig. 8) as a percentage of the maximum value obtained. The density is almost cylindrical symmetric, the deviation is small and can be assumed to be related to the orientation of the electric field. Even if the microwave electric field amplitude is almost symmetric, the electric field orientation is everywhere oriented with the Y direction. Close to the plasma chamber axis, there is a region of low density especially in correspondence of the extraction hole. It can be observed that the electrons tend to be far from the cylindrical walls starting from Z = 10 mm, in agreement with the focusing effect presented by Fig. 7.

### HSMDIS magnetic configuration

**Figure 9:**
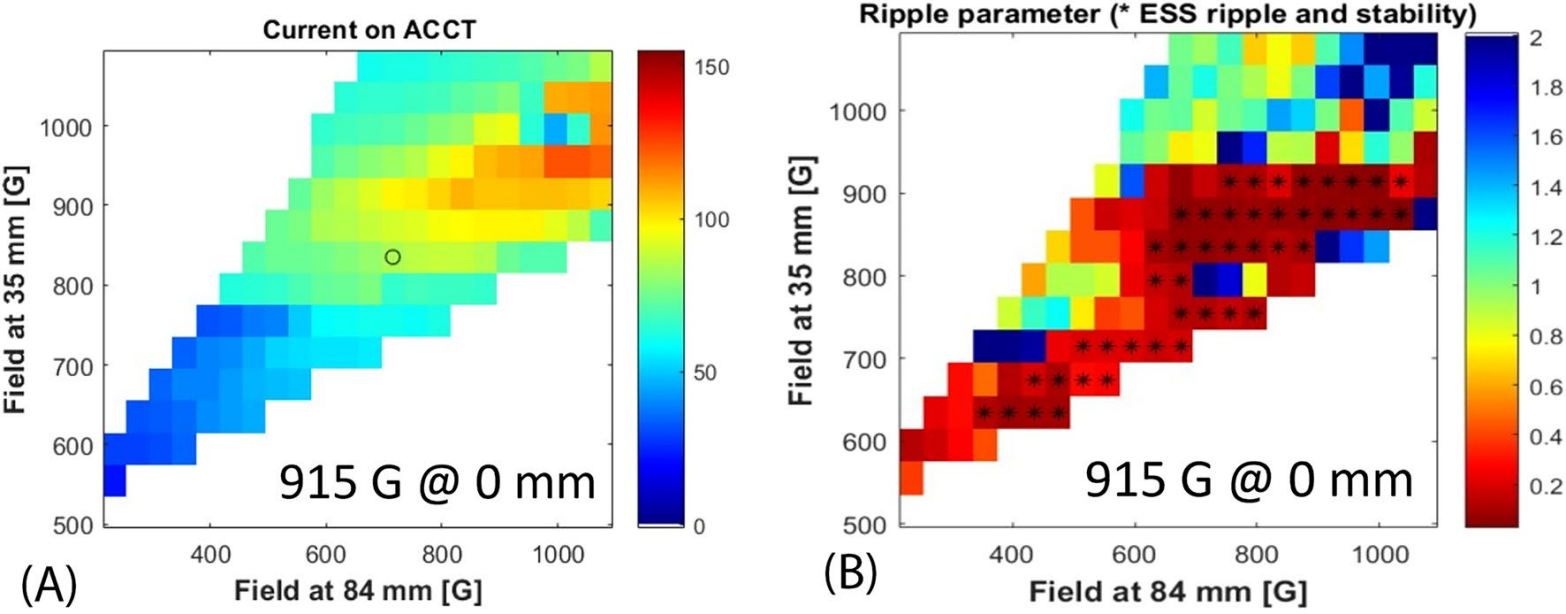
(**A**) ACCT current measured around the HSMDIS configurations (marked with a circle); (**B**) Ripple parameter map and ESS compliant configurations (marked with an asterisk).

**Figure 10:**
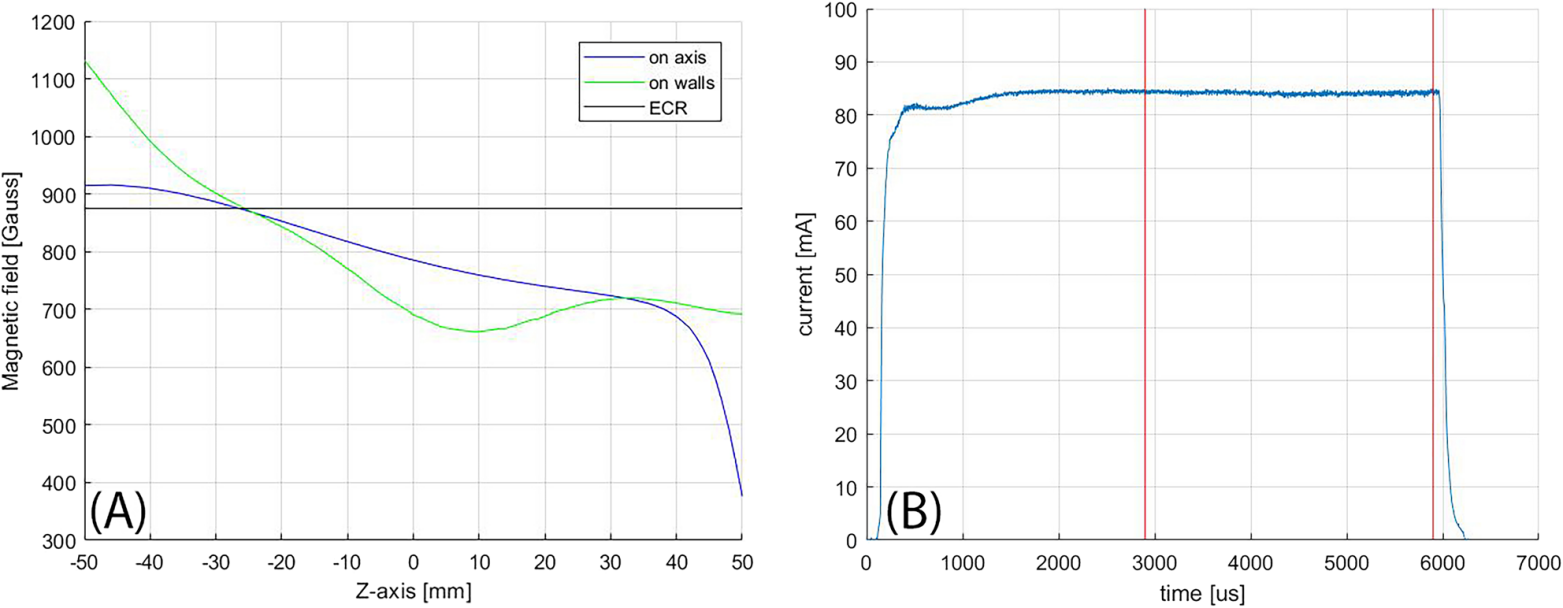
(**A**) Magnetic field configuration of HSMDIS configuration; (**B**) Corresponding beam pulse shape produced: only the beam produced within the red lines is transmitted to the accelerator.

HSMDIS magnetic configuration was found looking for stable beam production. A Ripple parameter was introduced in the analysis of the beam quality to identify the stable source magnetic configurations. The beam current over the pulse flat-top was fitted with a six-order polynomial fit then the standard deviation between the fit and the beam current values was named ripple-parameter. It evaluates how much the flat-top of the beam pulse differs from a smooth trend. By looking at this parameter (Fig. 9B) it is possible to identify a wide region of low noise beam production that perfectly catches all the configurations, marked with an asterisk, that satisfies the ESS beam stability requirements. It is interesting to note that there is no correlation between the high stability region and the trend of extracted beam current value (Fig. 9A). The centre of this stability region was named the HSMDIS magnetic configuration. It is in the following magnetic field coordinates: 915 G at 0 mm, 835 G at 35 mm, 715 G at 84 mm. The microwave power and the gas flux are the same as in the standard MDIS configuration (600 W and 3.0 SCCM). The magnetic configuration profiles and the beam current pulse shape produced is shown in Fig. 10. The beam current production reaches, after 2 ms, a high stability regime with no fluctuations and no descending or ascending trend. The magnetic field profile shows something unique, there is a crossing between the magnetic field profiles on-axis and the magnetic field profile on-walls exactly at the ECR value. This means that at this Z-coordinate the magnetic field is at the ECR value, everywhere, from the axis to the walls. In other words, the ECR value is distributed on a transverse slice of the plasma chamber.

**Figure 11:**
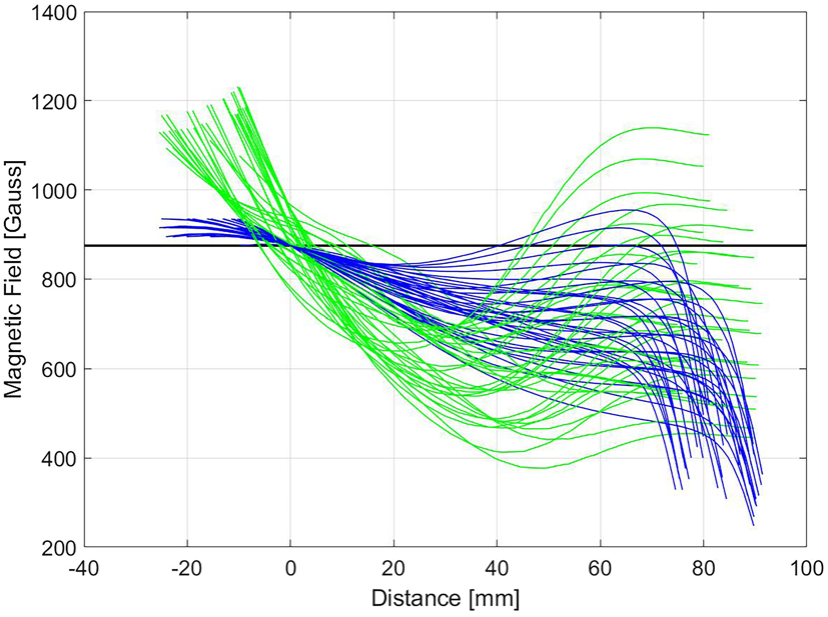
Superimposition of magnetic field profiles of different magnetic configurations of HSMDIS class.

A representative subset of magnetic configurations fully satisfying the ESS stability requirements are shown in Fig. 11. The complete set of magnetic configurations has been not shown to avoid the superposition of too many curves, therefore losing the capability to discern between the trends. The magnetic configuration profiles (on-axis and on-walls) were represented as a function of the distance from the ECR crossing of the on-axis magnetic field profile. This was done to observe that all the corresponding on-walls magnetic profiles cross the ECR value at a maximum distance of 10 mm from the on-axis ECR crossing (located at the origin). The HSMDIS configuration, selected as the centre of stability region, shows (Fig. 10A) an exact double ECR crossing, this confirms the correlation between stability and double ECR crossing. The small distance between the two ECR crossings of the other stable configurations, identify a little curvature of the ECR surface, sometimes versus the extraction or otherwise versus the injection. Another interesting similarity between stable configurations is that the magnetic field on the injection flange needs to be close to 915 Gauss, needed condition to enable the plasma heating in correspondence with the injection flange (Fig. 5). From the ECR crossing to the extraction region, a great variety of magnetic configurations are compatible with a stable beam production. Nevertheless, double ECR crossing is a necessary but not sufficient condition for stability. There are many magnetic configurations with double ECR crossing that don’t show high stability. A clear understanding of the new heating mechanism and plasma dynamics activated by the double ECR crossing is presented in the next section.

Not all configurations satisfying the ESS stability requirements fulfil also the ESS intensity requirement. The HSMDIS configuration satisfies both and shows a linear correlation (R^2^ = 0.9994) between the produced current and the injected microwave power: 79.2 mA at 550 W, 84.2 mA at 600 W, 88.8 mA at 650 W.

**Figure 12:**
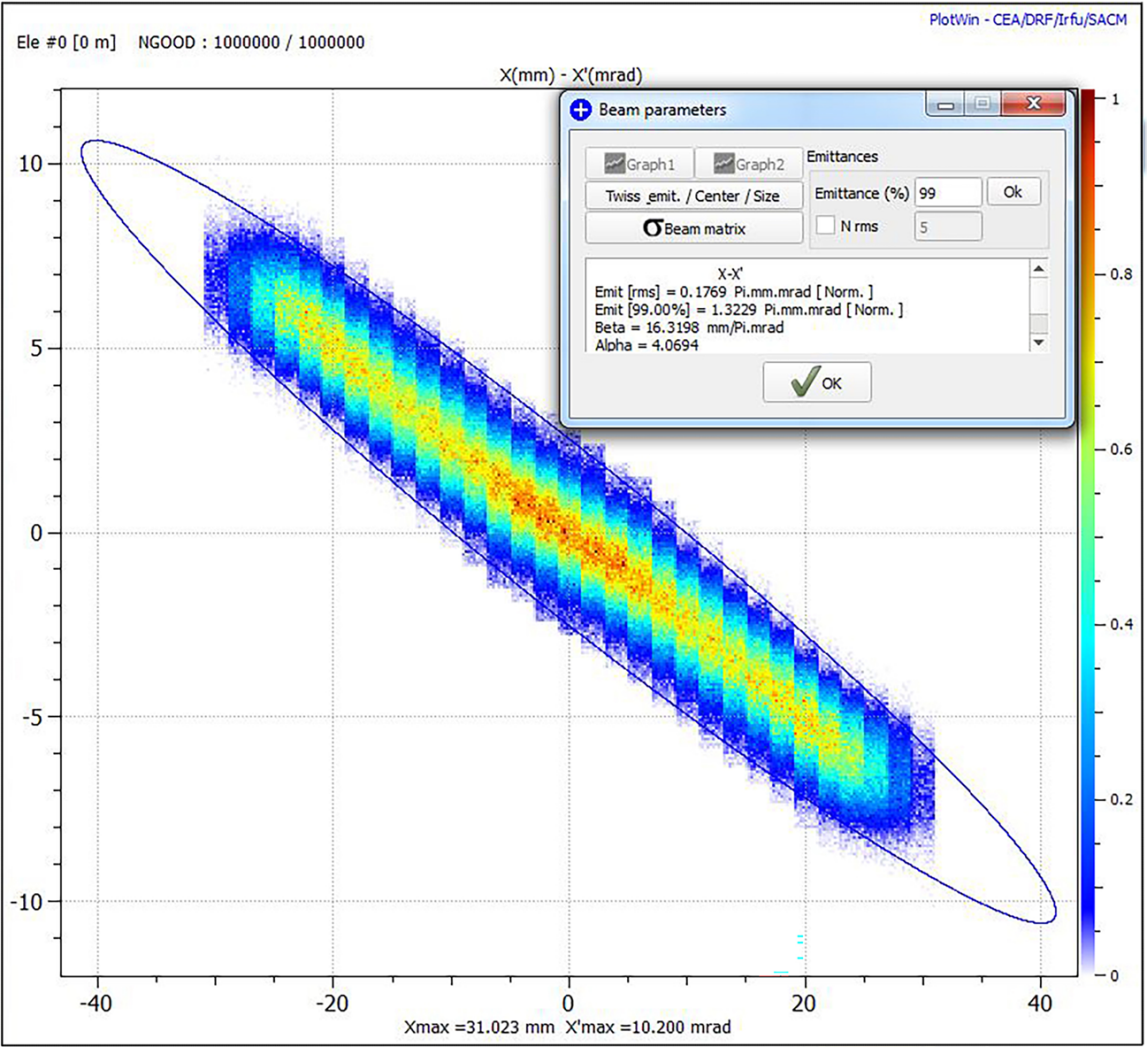
Beam emittance measurement of the beam produced with one of the most representative HSMDIS configurations (picture generated with PlotWin-CEA/IRFU).

After the beam stability and the amount of produced beam current, the most important beam parameter is the emittance. Unfortunately, each beam emittance measurement requires approximately one hour to be carried out, and the time scheduled for the PS-ESS commissioning at INFN-LNS was limited. These constraints have forbidden an exhaustive beam emittance characterization and only a few cases were analysed. Stable configurations, containing the double ECR crossing, produce a beam emittance lower than everything experienced with the standard MDIS configuration. An example is shown in Fig. 12 where the measured RMS beam emittance of 0.1769 π·mm·mrad, is two times lower than the 0.398 π·mm·mrad emittance measured, with the standard MIDS configuration, during the commissioning of the same source at Lund^25,26^.

### HSMDIS plasma simulation

**Figure 13:**
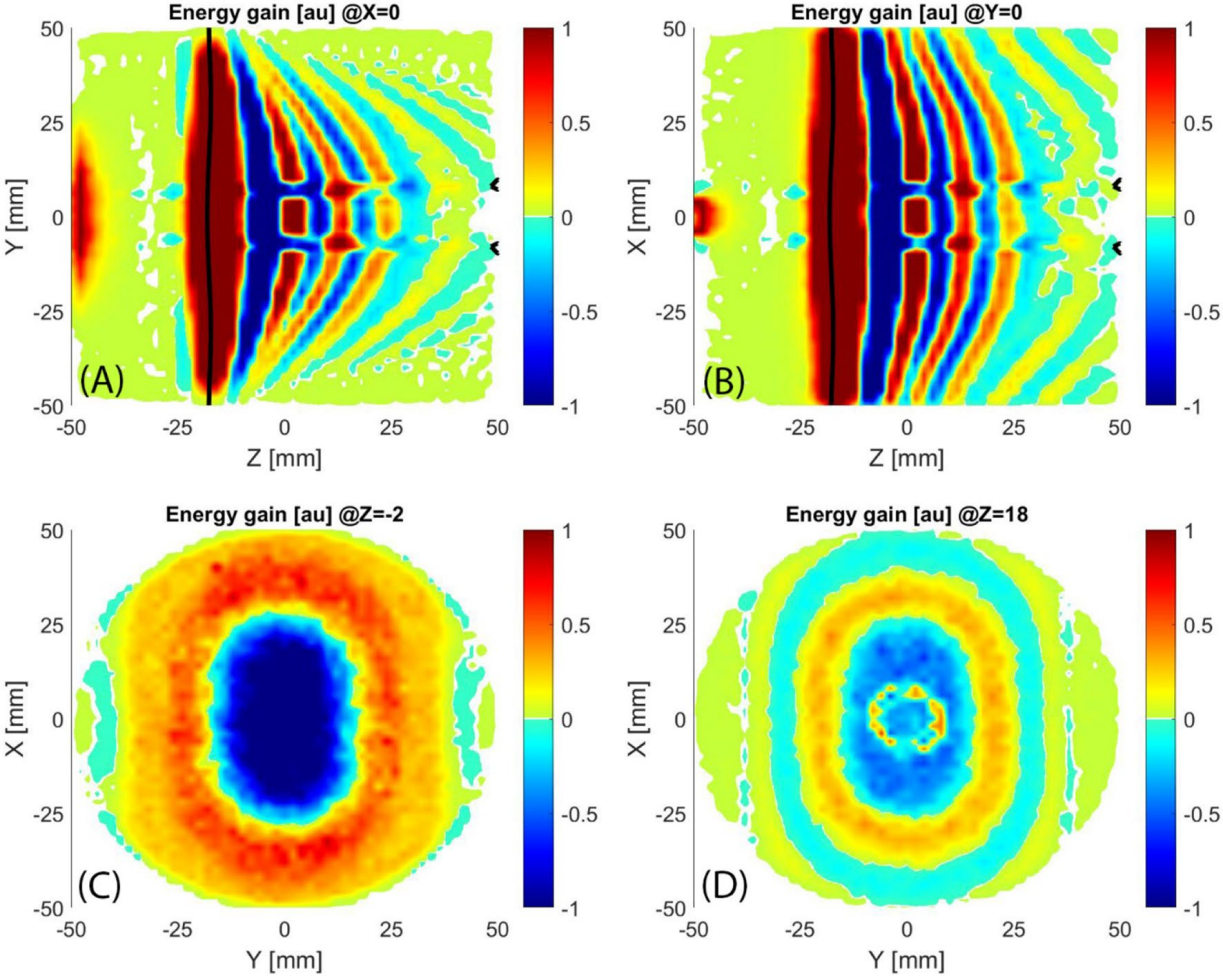
Longitudinal sections (**A**,**B**) and transversal sections (**C**,**D**) of the energy gain distribution, black lines mark the ECR regions.

Plasma simulation of HSMDIS configuration shows the generation of a new plasma heating mechanism (Fig. 13) defining a new class of MDIS. As expected, in correspondence of the ECR surface, highlighted with a black line in the longitudinal sections of Fig. 13, there is an intense RF energy transfer to the electrons. At the right of the ECR surface, there is a sequence of energy gain and energy loss regions. By looking at all sections of Fig. 13, it is possible to identify a dome shape with a curvature that increases with increasing the distance from the ECR surface.

**Figure 14:**
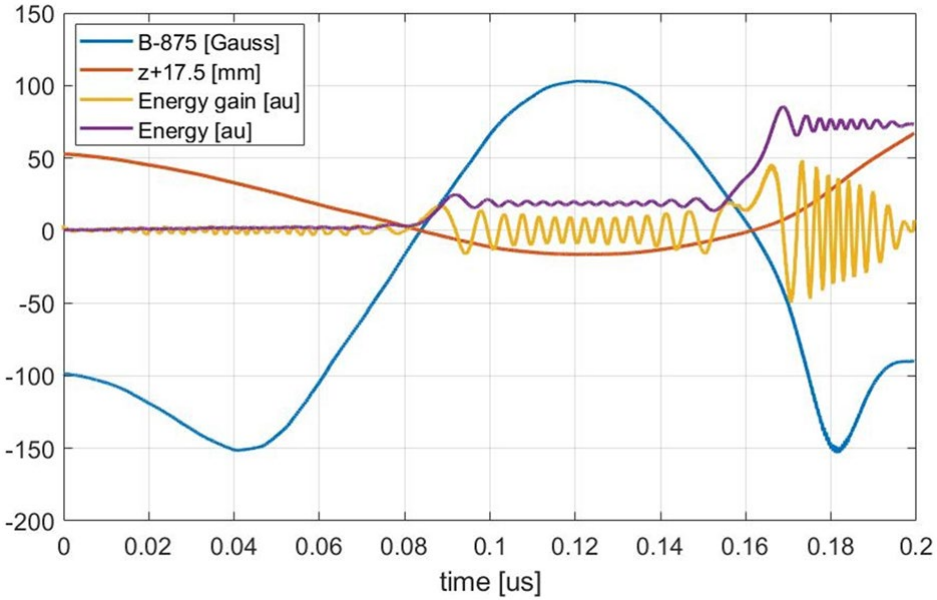
trajectory parameters of an electron that cross two times the ECR region.

The trajectory of one electron is analysed in Fig. 14 to investigate the reason for this alternation of acceleration and deceleration regions. The electrons in the solenoidal magnetic configuration move prevalently parallel to the axis of the plasma chamber with a helicoidal trajectory. Consequently, the axis coordinate z was chosen to identify the position of the electron inside the plasma chamber. The z coordinate was shifted by 17.5 mm to easily identify when the electron travelled across the ECR surface located at this coordinate. For the same reason, the magnetic field along the electron trajectory was instead shifted by 875 Gauss. This electron was born in the region close to the extraction (z ≈ 50 mm) and moves to the injection flange. After the cross of the ECR region reversed the z motion and came back to the extraction region where is lost on the wall. Energy and energy gain are shown to identify the contribution of this electron to acceleration and deceleration regions. As expected, the electron gain energy every time cross the ECR region. Less obvious is the alternation between accelerations and decelerations after the ECR crossing. Outside of the ECR region, the electron rotates around the magnetic field lines with a frequency that is close to the RF frequency, the relative difference is proportional to the difference between the magnetic field along the trajectory and the magnetic ECR value. The phase between these two oscillations changes slowly, so from when the electron is accelerated (phase = 0°), we observe the reduction of the energy gain sinusoidally proportionally to the phase increase. The phase increases up to 90° where no acceleration is observed, then start to be in phase-opposition (deceleration) up to arrive at 180° where can be observed the strongest deceleration. The further phase increase produces less deceleration up to bring back to the acceleration phase. The energy gain curve shows a fluctuation proportional to the electron energy and the electromagnetic field distribution. The strong energy gain at ECR forces the oscillation to start from a phase equal to zero in correspondence with the ECR region. This means synchronization of the energy gain oscillation for all electrons. The acceleration and deceleration regions in Fig. 13 came out from this synchronization. However, the difference between electrons velocity produces a worsening superposition of energy gain oscillation, resulting in an acceleration and deceleration region rapidly decreasing in intensity going far from ECR.

The described energy gain oscillation is a beats phenomenon with a frequency proportional to the difference between the magnetic field observed by the electron and the magnetic ECR value:

$$W_{beats} = \frac{{q_{e} \left( {B - B_{ECR} } \right)}}{{m_{e} }}$$

**Figure 15:**
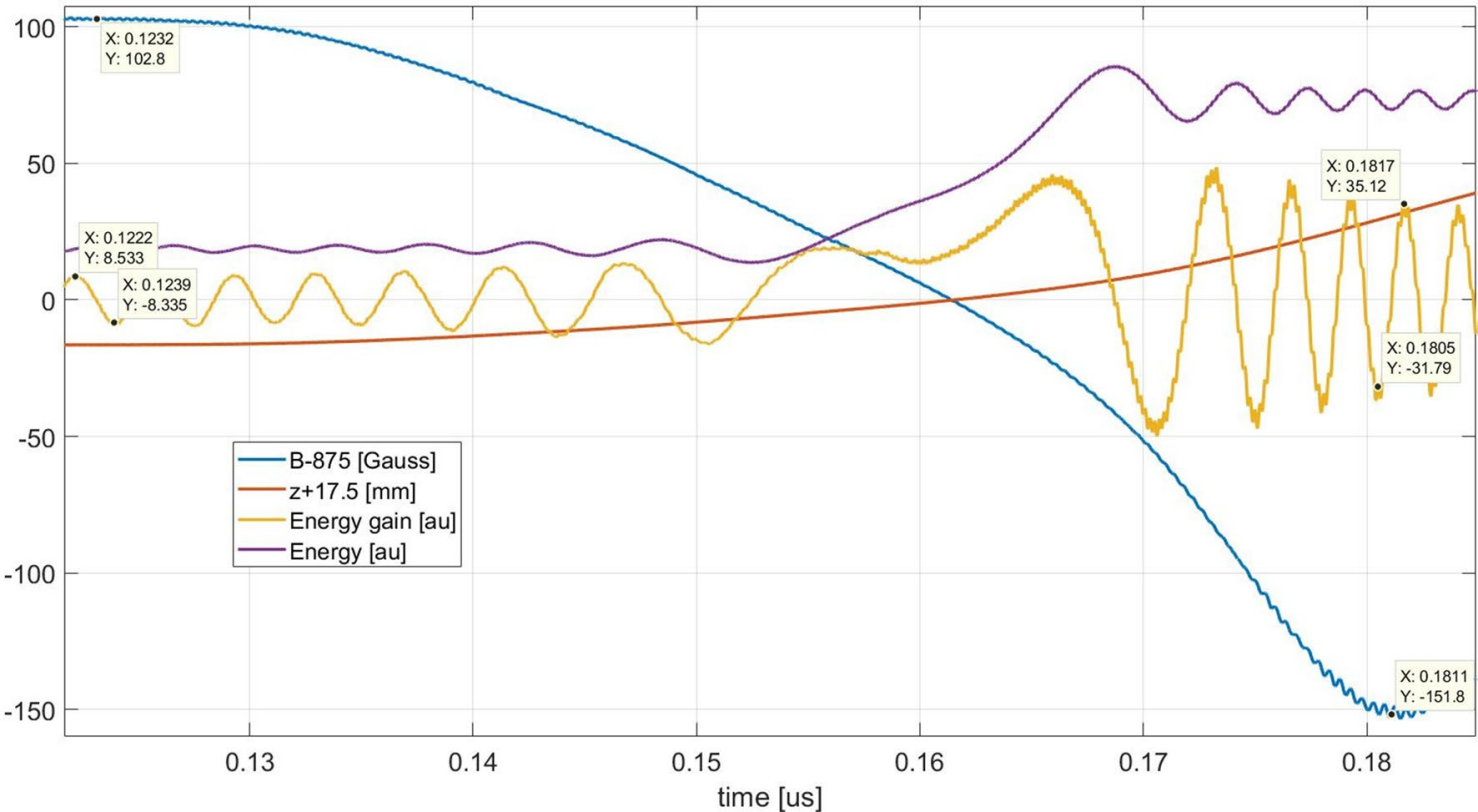
Beats analysis.

Excellent confirmation of this assertion came out from the analysis of some details of the electron trajectory (Fig. 15). At the magnetic field of ECR +102.8 G, corresponding to a beat frequency of 287.84 MHz, the half period is 123.94–122.2 = 1.74 ns equivalent to a frequency of 287.36 MHz. At the magnetic field of ECR −51.752 G, corresponding to a beat frequency of 424.91 MHz, the half period is 181.665–180.49 = 1.175 ns equivalent to a frequency of 425.53 MHz.

**Figure 16:**
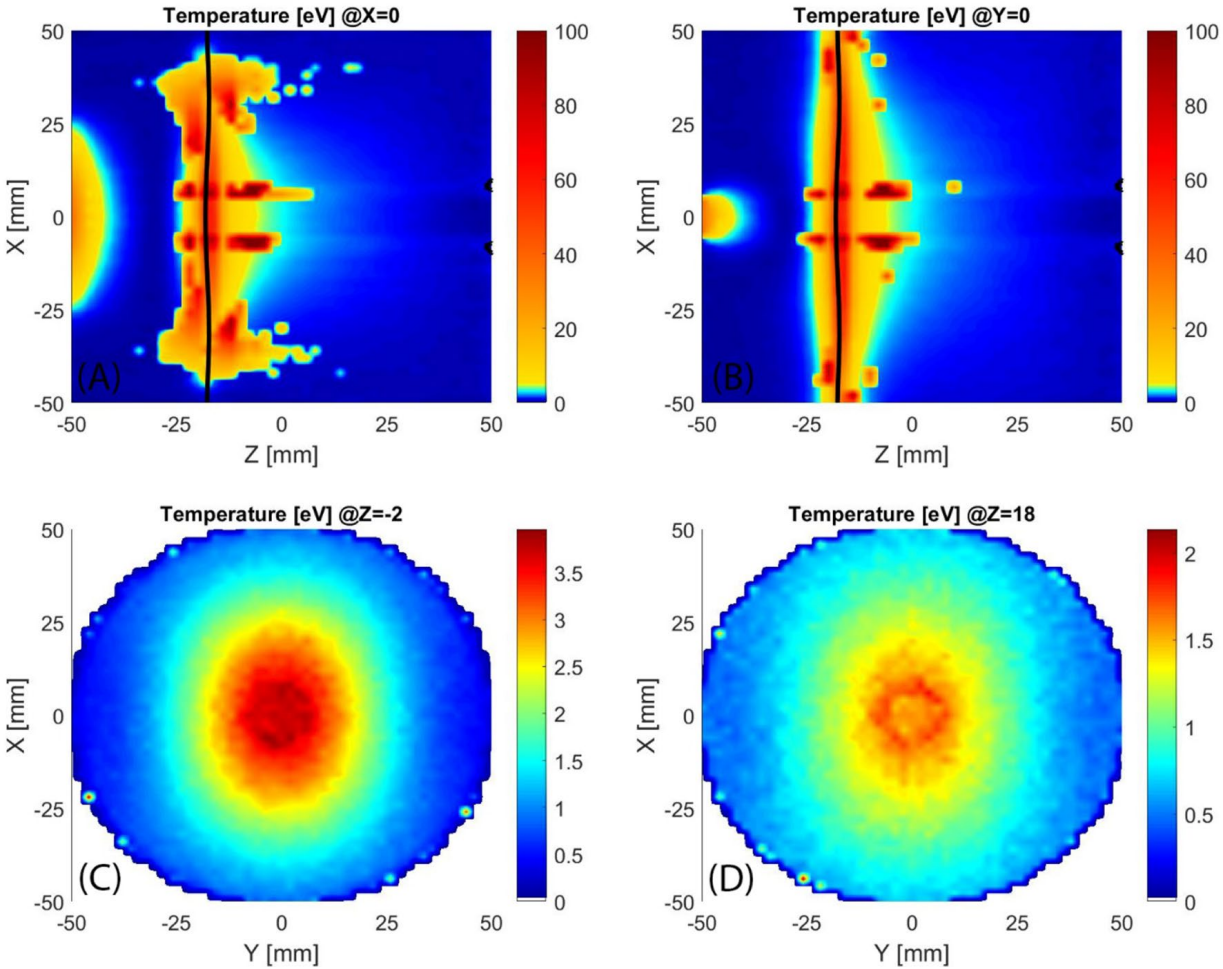
Longitudinal sections (**A**,**B**) and transversal sections (**C**,**D**) of evaluated electron temperature spatial distribution, black lines mark the ECR regions.

In correspondence with the injection flange, the EESDF shows (Fig. 16 with the same colour-map strategy of Fig. 6) the same peak observed in the simulation of standard MDIS magnetic configuration. This is in agreement with the same energy gain presented in Figs. 5 and 13. In correspondence with the ECR region, the heating is intense and temperatures rise to 100 eV. After the ECR acceleration, almost all electrons move rapidly to the extraction flange, where they are almost lost, with a speed proportional to their energy. Consequently, the contribution to the EESDF of more energetic electrons weighs less than lower-energy electrons and the evaluated temperature is lower than what is observed in the ECR region. A relative hotter distribution is observed in correspondence of the axis where the electrons bunch back, as presented in Fig. 18.

**Figure 17:**
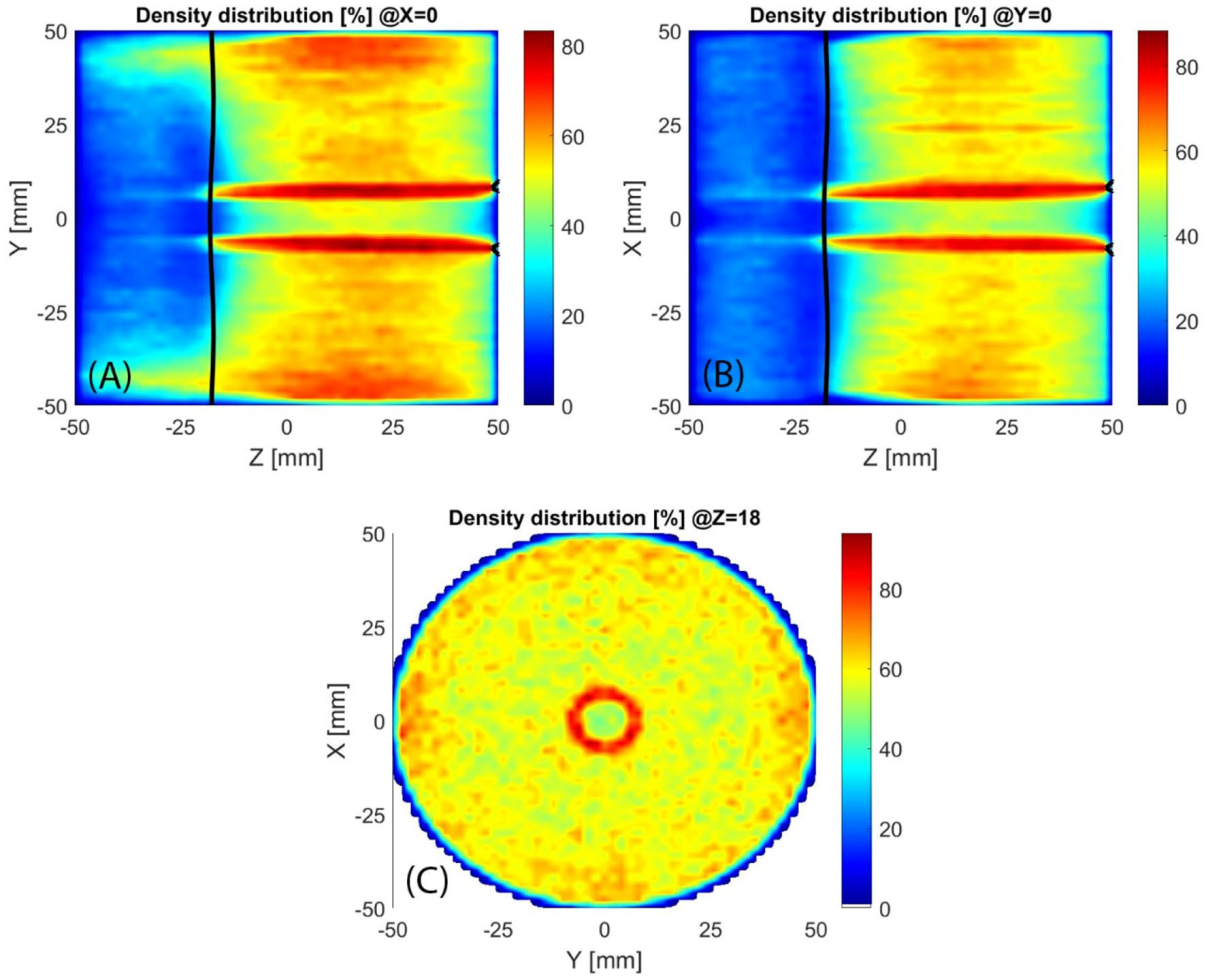
Longitudinal sections (**A**,**B**) and transversal section (**C**) of electron density, black lines mark the ECR regions.

The analysis of the electron density distribution (Fig. 17) shows that the plasma is prevalently located in between the ECR region and the extraction flange. This agrees with the higher current extracted from the standard MDIS magnetic configuration that fills all the volume of the plasma chamber instead of the half-filled by the HSMDIS configuration. A cylindrical higher density region is located at the same diameter of the extraction hole where there is a small ECR region (black lines close to the extraction hole). This small ECR region is due to the ferromagnetic shielding located inside the extraction flange.

**Figure 18:**
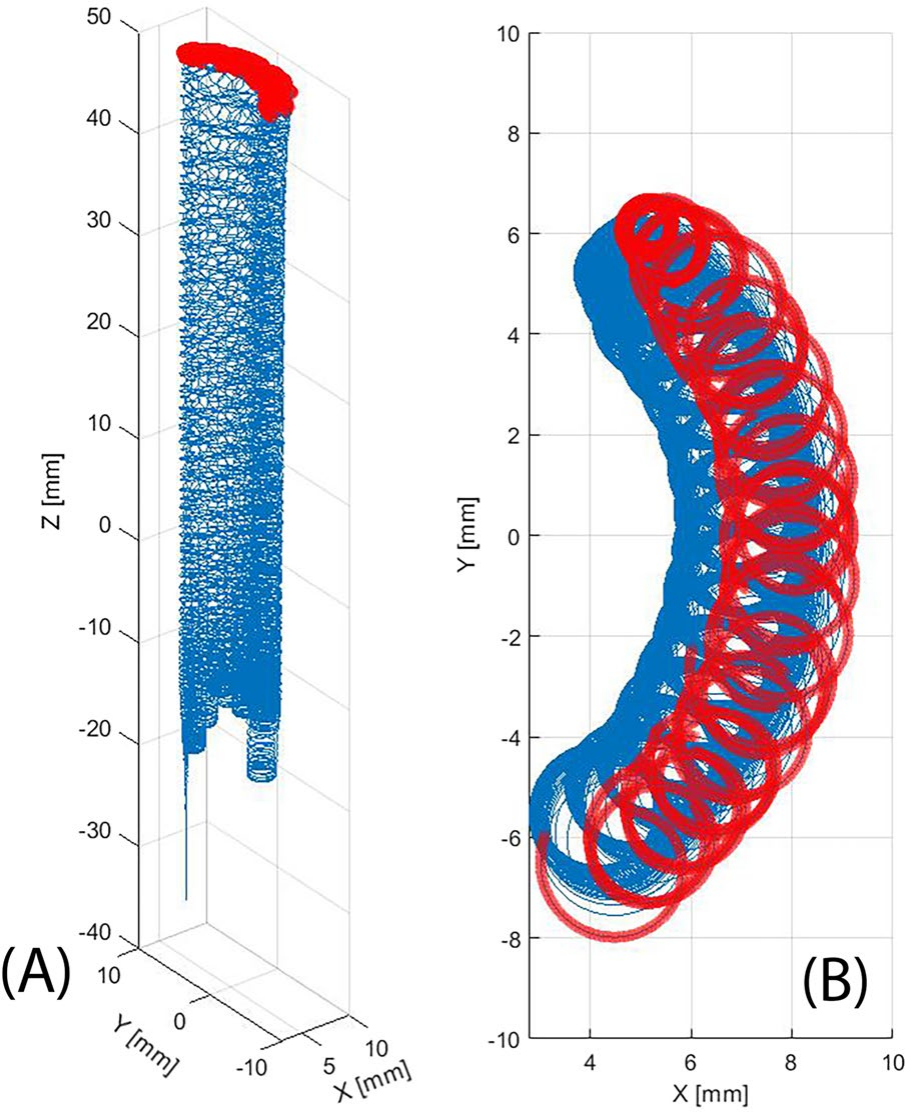
Three-dimensional view (**A**) and z-axis view (**B**) of one electron takes part of the cylindrical high-density region, trajectory closer to the extraction hole was marked with red.

The trajectory of electrons contributing to this high-density cylindrical region shows that the extraction hole ECR region can axially confine electrons. The trajectory of one of them is shown in Fig. 18. The electron goes several times back and forth, from the central ECR region to the extraction hole ECR region, with a helicoidal trajectory. The path close to the extraction hole, marked with red, shows that every time the motion direction is reversed the trajectory is subject to a precession along the extraction hole diameter. The precession diameter is approximately 7 mm while the extraction hole diameter is 8 mm.

## Discussion and perspectives

The plasma density obtained with MDIS increase up to the cut-off density, because over the cut-off density the RF microwaves propagation inside the plasma is strongly attenuated. The microwaves are responsible for the plasma heating process and at the same time, the plasma generated acts as shielding for microwave propagation inside the plasma chamber. These two competitive processes generate plasma stability. When equilibrium is obtained, a hypothetical increase of density produces the increase of microwave shielding and consequently a reduction of new plasma particles generation, and vice versa, a reduction of plasma density reduce the shielding and the additional incoming RF power will increase the generation of new plasma particles. The stability strength is proportional to the intensity of the counteracting phenomena. Close to the RF injection flange, the magnetic field of the HSMDIS configuration (Fig. 10A) is approximately the same as in the standard MDIS configuration (Fig. 4A), and the produced energy gain distribution has the same characteristics (Figs. 5 and 13). However, the plasma density distributions of the two magnetic configurations (Figs. 8 and 17) are completely different. The high plasma density region of standard MDIS configuration is in touch with this heating region while the high plasma density of HSMDIS configuration is far. The main plasma heating region of the HSMDIS configuration is the ECR surface at z = 17.5 cm that is in touch with the high plasma density region of this magnetic configuration. In the standard MDIS configuration, the plasma heating region is close to the injection wall while the high plasma density cannot be in touch with the walls, so the shielding effect is limited. Instead, in the HSMDIS configuration, the high plasma density region can expand over the ECR region, and so can switching off the plasma heating. This represents a strong interaction between the two counteracting phenomena and is the reason for the high stability of the HSMDIS magnetic configuration. The divergence and the spatial density distribution of the extracted beam are related to the plasma density directly and indirectly through the shape of the plasma meniscus that depends on the plasma density. The high stability of the HSMDIS configuration ensures a little plasma parameters variation during the beam extraction (Fig. 10B), the beam emittance is a time-integrated measurement of beam parameters and consequently, small beam parameter variation produces low beam emittance (Fig. 12). Comparing the plasma density distribution of the two simulations (Figs. 8 and 17) it is also possible to notice that the volume occupied by plasma in HSMDIS configuration is approximately 67% of the space occupied in MDIS configuration, approximately the same ratio is found considering the value of extracted beam current (Figs. 4B and 10B), 135 mA vs 83 mA (61%). The difference can be attributed to the stronger shielding capability of the plasma in the HSMDIS configuration, able to move the equilibrium to lower plasma density.

The high degree of freedom inserted in the design of the PS-ESS magnetic system was the most important innovation introduced in the source design. The use of physics correlated parameters, the capability to test several tens-thousands of source configurations and the definition of a ripple parameter permitted the identification of a completely new class of magnetic configurations (HSMDIS) enabling the production of high-stability and low emittance beams. Our Stationary-PIC simulation strategy revealed all his potentiality making us able to understand the new plasma heating mechanism and why this new magnetic configuration produces high stability plasma and low emittance extracted beam. The excellent performance improvements observed and the capability to simulate plasma dynamics will permit access to higher-current sources development. The simulation tool will be developed trying to reach the self-consistency will make able to simulate the extracted beam. The same development strategy will be applied to the development of high charge state ECR ion sources.

## References

[1] Geller R (1976). Electron cyclotron resonance multiply charged ion sources. IEEE Trans. Nucl. Sci..

[2] Arianer J, Geller R (1981). The advanced positive heavy ion sources. Annu. Rev. Nucl. Part. Sci..

[3] Geller R (1990). ECRIS: The electron cyclotron resonance ion sources. Annu. Rev. Nucl. Part. Sci..

[4] Geller R (1998). Electron cyclotron resonance sources: Historical review and future prospects. Rev. Sci. Instrum..

[5] Taylor T, Willis JSC (1991). A high-current low-emittance dc ECR proton source. Nucl. Instrum. Methods Phys. Res. A.

[6] Grolleau B (1974). Study of the characteristics of the ion beam extracted from an rf discharge in hydrogen under conditions of resonance. J. Appl. Phys..

[7] Sakudo N, Tokiguchi K, Koike H, Kanomata I (1977). Microwave ion source. Rev. Sci. Instrum..

[8] Miracoli R (2012). Characterization of microwave discharge ion source for high proton beam production. Eur. Phys. J. Plus.

[9] Celona L, Neri L, Gammino S (2018). High intensity proton source and LEBT for the European spallation source. Proceedings of the 17th International Conference on Ion Sources. AIP Conf. Proc..

[10] Neri L (2017). Beam commission of the high intensity proton source developed at INFN-LNS for the European Spallation Source, 8th International Particle Accelerator Conference. J. Phys. Conf. Ser..

[11] Neri L (2014). Improved design of proton source and low energy beam transport line for European Spallation Source. Rev. Sci. Instrum..

[12] Celona, L. *et al*. Ion source and low energy beam transport line final commissioning step and transfer from INFN to ESS. *9th International Particle Accelerator Conference.*10.18429/JACoW-IPAC2018-TUPML073 (2018).

[13] Celona, L. *et al*. Design issues of the proton source for the ESS facility. LINear Accelerator Conference, THPB076, ISBN 978-3-95450-122-9, pp. 1008–1010 (2012).

[14] Torrisi G (2017). Microwave injection and coupling optimization in ECR and MDIS ion sources. 8th International particle accelerator conference. J. Phys. Conf. Ser..

[15] Neri, L. *et al*. High level control system code with automatic parametric characterization capabilities. *16th International Conference on Accelerator and Large Experimental Physics Control Systems.*10.18429/JACoW-ICALEPCS2017-THPHA035 (2017).

[16] Neri, L. *et al*. Commissioning of the high intensity proton source developed at INFN-LNS for the European Spallation Source. *LINear Accelerator Conference*, MOPLR057, ISBN 978-3-95450-169-4, pp. 261–263 (2016).

[17] Mascali D (2010). Plasma ion dynamics and beam formation in electron cyclotron resonance ion sources. Rev. Sci. Instrum..

[18] Neri L (2012). 3D Monte Carlo code for the modeling of plasma dynamics and beam formation mechanism in electron cyclotron resonance ion sources. Rev. Sci. Instrum..

[19] Neri L (2016). Recent progress in plasma modelling at INFN-LNS. Rev. Sci. Instrum..

[20] Boris, J. P. *et al*. Relativistic plasma simulation-optimization of a hybrid code. *Proceedings of the 4th Conference on Numerical Simulation of Plasmas* 3–67 (Naval Res. Lab., 1970)

[21] Roychowdhury P, Chakravarthy DP (2009). High intensity electron cyclotron resonance proton source for low energy high intensity proton accelerator. Rev. Sci. Instrum..

[22] Woods, L. C. *Principles of Magnetoplasma Dynamics* (Oxford University Press, 1987). ISBN10 0198562209

[23] Caruso A (2018). Experimental performance of the chopper for the ESS LINAC, 9th International Particle Accelerator Conference. J. Phys. Conf. Ser..

[24] Neri, L. *et al*. The ESS low energy beam transport line design. Linear Accelerator Conference, THPB028, ISBN 978-3-95450-122-9, pp. 912–914 (2012).

[25] Eshraqui M (2020). First protons in the ESS LINAC. J. Surf. Investig..

[26] Miyamoto, R. *et al*. First results of beam commissioning on the ESS site for the ion source and low energy beam transport. 10th International Particle Accelerator Conference. 10.18429/JACoW-IPAC2019-MOPTS103 (2019).

