# Author Correction: High stability microwave discharge ion sources

**DOI:** 10.1038/s41598-022-09667-y

**Published:** 2022-04-05

**Authors:** L. Neri, L. Celona

**Affiliations:** INFN-Laboratori Nazionali del Sud, via S. Sofia 62, 95123 Catania, Italy

Correction to: *Scientific Reports* 10.1038/s41598-022-06937-7, published online 23 February 2022

The original version of this Article contained errors.

In Table 1, the Parameter values were shifted down by one row. As a result, the values for “RF power” were omitted. The incorrect and correct values appear below.

Incorrect:

**Table:** 

Parameter		
Magnetic field at 0 mm	Min	Max
Magnetic field at 35 mm	795 G	1015 G
Magnetic field at 84 mm	515 G	1395 G
H2 flow	235 G	1995 G
RF power	2 SCCM	5 SCCM

Correct:

**Table:** 

Parameter	Min	Max
Magnetic field at 0 mm	795 G	1015 G
Magnetic field at 35 mm	515 G	1395 G
Magnetic field at 84 mm	235 G	1995 G
H2 flow	2 SCCM	5 SCCM
RF power	550 W	650 W

In addition, the original version of this Article contained errors in the text.

In the Experimental setup section, under the subheading ‘Standard MDIS magnetic configuration’,

“This area is evident in Fig. 2 where ACCT current is shown as a function of the magnetic field at 35 mm and 84 mm, these data are the subset of measurement taken with 915 Gauss at 0 mm from the injection flange, 600 W of injected microwave power and 3.0 Standard Cubic Centimetres per minute (SCCM) of H2 gas flux.”

now reads:

“This area is evident in Fig. 3 where ACCT current is shown as a function of the magnetic field at 35 mm and 84 mm, these data are the subset of measurement taken with 915 Gauss at 0 mm from the injection flange, 600 W of injected microwave power and 3.0 Standard Cubic Centimetres per minute (SCCM) of H2 gas flux.”

The first subheading ‘Plasma simulation’ has been renamed to ‘MDIS plasma simulation’.

Under the subheading ‘MDIS plasma simulation’,

“The little energy gain, uniformly distributed in the rest of the plasma chamber, is due to energy exchange, by Coulomb collisions, with the background plasma that was initialized with 2 eV temperature distribution. No energy gain is observed in the ECR region close to the extraction hole due to a very low intensity of the microwave electric field in this region. An evaluation of the Electron Energy and Spatially Distribution Function (EESDF) may be carried out by storing the energy of electrons passing through each cell. With a Maxwellian fit, it is possible to extract the temperature spatial distribution (Fig. 5). In the longitudinal sections (5A and 5B) the colour scale is divided into two parts: the first part (from blue to green) is used for temperatures ranging from 0 eV to 5% (1.9 eV) of the maximum evaluated temperature (38 eV), while the latter part (from yellow to red) is used from 5 to 100%. In the two transversal views (5C and 5D), obtained at two different longitudinal positions (Z =  − 2 mm and Z = 18 mm) the colour scale is uniform because there is only a warm population.”

now reads:

“The little energy gain, uniformly distributed in the rest of the plasma chamber, is due to energy exchange, by Coulomb collisions, with the background plasma that was initialized with 6 eV temperature distribution. No energy gain is observed in the ECR region close to the extraction hole due to a very low intensity of the microwave electric field in this region. An evaluation of the Electron Energy and Spatially Distribution Function (EESDF) may be carried out by storing the energy of electrons passing through each cell. With a Maxwellian fit, it is possible to extract the temperature spatial distribution (Fig. 6). In the longitudinal sections (6A and 6B) the colour scale is divided into two parts: the first part (from blue to green) is used for temperatures ranging from 0 eV to 5% (1.9 eV) of the maximum evaluated temperature (38 eV), while the latter part (from yellow to red) is used from 5 to 100%. In the two transversal views (6C and 6D), obtained at two different longitudinal positions (Z =  − 2 mm and Z = 18 mm) the colour scale is uniform because there is only a warm population.”

Under the subheading ‘MDIS plasma simulation’, the following sentence was removed:

“A complementary view of the temperature distribution is offered by the two transversal slices presented in Fig. 6.”

Under the subheading ‘HSMDIS magnetic configuration’,

“A new HSMDIS magnetic configuration was found looking for stable beam production.”

now reads:

“HSMDIS magnetic configuration was found looking for stable beam production.”

“Another interesting similarity between stable configurations is that the magnetic field on the injection flange needs to be close to 915 Gauss, needed condition to enable the plasma heating in correspondence with the injection flange (Fig. 6).”

now reads:

“Another interesting similarity between stable configurations is that the magnetic field on the injection flange needs to be close to 915 Gauss, needed condition to enable the plasma heating in correspondence with the injection flange (Fig. 5).”

In addition, the second subheading ‘Plasma simulation’ has been renamed to ‘HSMDIS plasma simulation’.

Furthermore, the original version of this Article contained an error in Figure 4B where the data of the line graph was incorrect. The original Figure 4 and accompanying legend appear below.

**Figure 4:**
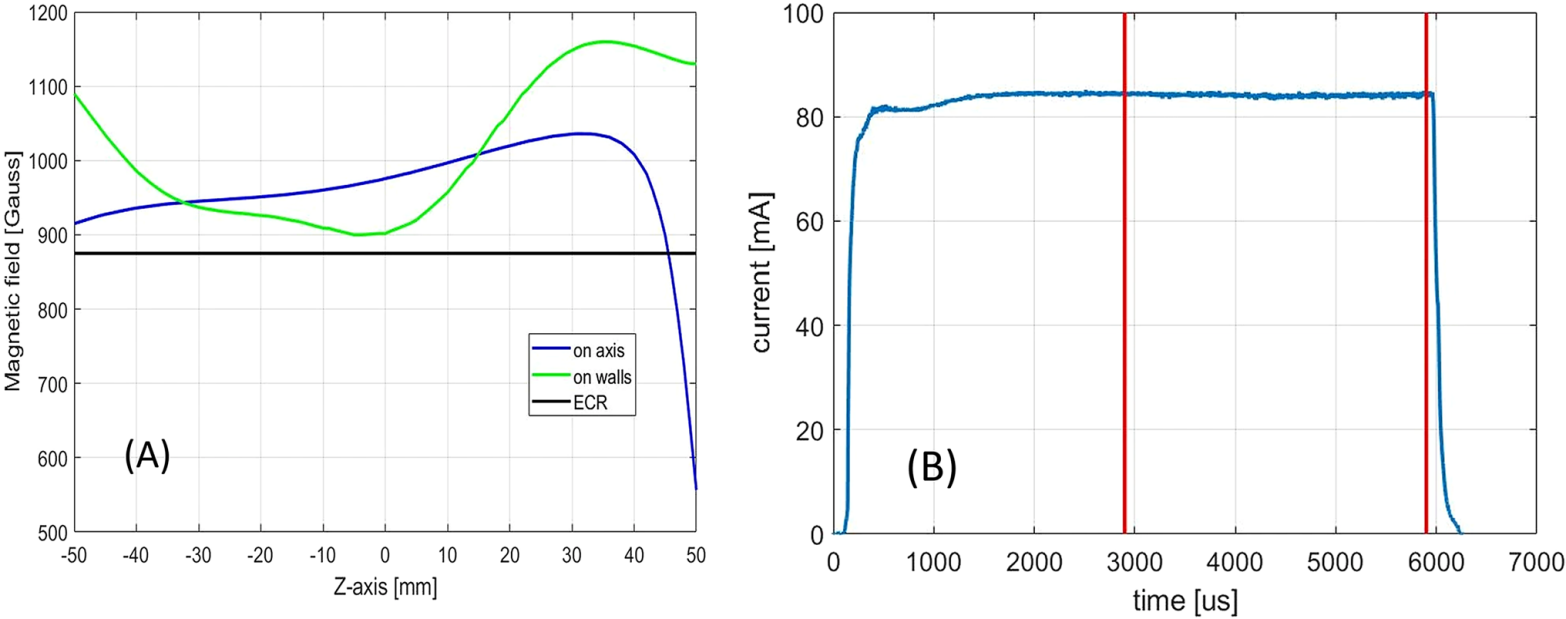
(**A**) Magnetic field configuration at the centre of the high current region; (**B**) correspondent beam pulse shape produced: only the beam produced within the red lines is transmitted to the accelerator.

The original Article has been corrected.

